# The immunological characteristics and probiotic function of recombinant *Bacillus subtilis* spore expressing *Clonorchis sinensis* cysteine protease

**DOI:** 10.1186/s13071-016-1928-0

**Published:** 2016-12-19

**Authors:** Zeli Tang, Mei Shang, Tingjin Chen, Pengli Ren, Hengchang Sun, Hongling Qu, Zhipeng Lin, Lina Zhou, Jinyun Yu, Hongye Jiang, Xinyi Zhou, Xuerong Li, Yan Huang, Jin Xu, Xinbing Yu

**Affiliations:** 1 Department of Parasitology, Zhongshan School of Medicine, Sun Yat-sen University, 74 Zhongshan 2nd Road, Guangzhou, 510080 China; 2 Key Laboratory for Tropical Diseases Control, Sun Yat-sen University, Ministry of Education, Guangzhou, 510080 Guangdong China

**Keywords:** *Clonorchis sinensis*, *Bacillus subtilis* spore, Cysteine protease, Oral immunization, Immunological characteristics

## Abstract

**Background:**

Clonorchiasis, a food-borne zoonosis, is caused by *Clonorchis sinensis*. The intestinal tract and bile ducts are crucial places for *C. sinensis* metacercariae to develop into adult worms. The endospore of *Bacillus subtilis* is an ideal oral immunization vehicle for delivery of heterologous antigens to intestine. Cysteine protease of *C. sinensis* (*Cs*CP) is an endogenous key component in the excystment of metacercariae and other physiological or pathological processes.

**Methods:**

We constructed a fusion gene of CotC (a coat protein)-*Cs*CP and obtained *B. subtilis* spores with recombinant plasmid of pEB03-CotC-*Cs*CP (*B.s*-CotC-*Cs*CP). CotC-*Cs*CP expressed on spores’ surface was detected by Western blotting and immunofluorescence. Immunological characteristics of recombinant spore coat protein were evaluated in a mouse model. The levels of *Cs*CP-specific antibodies were detected by ELISA. Effects of recombinant spores on mouse intestine were evaluated by histological staining. The activities of biochemical enzymes in serum were assayed by microplate. Liver sections of infected mice were evaluated by Ishak score after Masson’s trichrome.

**Results:**

The *B.s*-CotC-*Cs*CP spores displayed *Cs*CP on their coat. Specific IgG and isotypes were significantly induced by coat proteins of *B.s*-CotC-*Cs*CP spores after subcutaneous immunization. IgA levels in intestinal mucus and bile of *B.s*-CotC-*Cs*CP orally treated mice significantly increased. Additionally, more IgA-secreting cells were observed in enteraden and *lamina propria* regions of the mouse jejunum, and an increased amount of acidic mucins in intestines were also observed. There were no significant differences in enzyme levels of serum among groups. No inflammatory injury was observed in the intestinal tissues of each group. The degree of liver fibrosis was significantly reduced after oral immunization with *B.s*-CotC-*Cs*CP spores.

**Conclusions:**

*Bacillus subtilis* spores maintained the original excellent immunogenicity of *Cs*CP expressed on their surface. Both local and systemic specific immune responses were elicited by oral administration of *B.s*-CotC-*Cs*CP spores. The spores effectively promoted intestinal health by inducing secretion of acidic mucins, with no other side effects to the liver or intestine. Oral administration of spores expressing *Cs*CP could provide effective protection against *C. sinensis*. This study may be a cornerstone for development of antiparasitic agents or vaccines against clonorchiasis based on *B. subtilis* spore expressing *Cs*CP on the surface.

**Electronic supplementary material:**

The online version of this article (doi:10.1186/s13071-016-1928-0) contains supplementary material, which is available to authorized users.

## Background

Clonorchiasis caused by *Clonorchis sinensis* is recognized as an important emergent or re-emergent human food-borne parasitic disease, one of the most common zoonoses in East Asia. Humans or animals can be infected mainly due to ingestion of raw or undercooked freshwater fish containing encysted metacercaria of *C. sinensis* [1, 2]. Metacercariae excyst in the duodenum of the host, then migrate into the bile duct, and further develop into adult worms [2]. Mechanical irritation, immunopathological processes and DNA damage caused by *C. sinensis* can induce hyperplasia of the bile duct epithelium and connective tissue and cause jaundice, indigestion, biliary inflammation and bile duct obstruction, even cholangiocarcinoma (CCA), liver cirrhosis and liver cancer in humans [1, 3]. Clonorchiasis has become a severe disease burden and brought serious medical and economic problems to the low- or middle-income countries of East Asia. It is estimated that more than 200 million people are threatened by *C. sinensis* infection, and over 15 million people are infected worldwide [2, 4]. The global burden of clonorchiasis is nearly 275,370 disability adjusted life years (DALYs), and 5,591 people have died from this infection every year [4]. It is urgent that effective prevention strategies such as vaccine trials, the development of antiparasitic agents and new health education be implemented.

Improvement of mucosal immunity is very important in conferring protection against pathogens (e.g. internal parasites) that typically invade *via* mucosal system [5, 6]. Oral immunization, for example, is a very straightforward, inexpensive and needle-free approach to deliver a vaccine to the mucosal lining of the gut and elicit protective immunity within the gut mucosa. However, oral immunization suffers from degradation by gastric acid and proteolysis in gastrointestinal tract, which generates a poor immune response [6, 7]. Therefore, effective heterologous antigen carriers should be chosen to solve the problem of limited absorption and tolerance in the gut.

A series of reports indicated that the endospore of *Bacillus subtilis* is an ideal vehicle for delivery of heterologous antigens to the gastrointestinal tract. First, spore-forming *B. subtilis* is a non-pathogenic and non-invasive aerobic Gram-positive bacterium [8, 9]. Spores of various *Bacillus* species are currently being used as probiotics and food supplements in both humans and animals [9, 10]. They can survive under extreme temperature, desiccation, pH and exposure to noxious chemicals and solvents [11, 12]. In addition, these spores possess convenient gene operability. Heterologous antigens can be stably and sufficiently displayed on the surface of spores using the outer coat proteins of the *B. subtilis* spore (such as CotB, CotC and CotG) as the fusion partner [12, 13]. In our laboratory, the oral immunization delivery platform based on a *B. subtilis* spore-engineering system has been successfully constructed and is proven to be valid and feasible [14–16].

The cysteine protease superfamily of parasite organisms plays a key role in physiology and related pathobiology processes that are closely related to larval migration, nutrition acquisition, egg hatching and immune evasion [3, 17–19]. Currently, cysteine proteases identified from various parasites (e.g. *C. sinensis*, *Fasciola hepatica*, *Taenia solium* and *Ancylostoma caninum*) have been exploited as major potential molecules for serodiagnosis, immuno-/chemo-therapy and vaccine candidates [19–25]. The cysteine protease from *C. sinensis* (*Cs*CP) is an important component of excretory-secretory products (ESPs) in both adult worms and metacercariae [19, 26]. Additionally, *Cs*CP is an indispensable protease participating in the excystment of metacercariae, enabling larvae to successfully move to bile ducts, and further develop into adults [26]. Therefore, *Cs*CP will be a potential target for effective vaccines and drug development.

In the present report, the encoding sequence of *Cs*CP was transformed into *B. subtilis* (WB600) using an *Escherichia coli*/*B. subtilis* shuttle vector. The CotC-*Cs*CP fusion gene was constructed and expressed on spore coat. Mice were administered subcutaneously with coat proteins extracted from CotC-*Cs*CP*-*transformed *B. subtilis* (*B.s*-CotC-*Cs*CP) spores or recombinant *Cs*CP protein purified from an *E. coli* expression system (r*Cs*CP). The evoked immune reactions were compared. Additionally, the specific local and systemic immune responses triggered by *B.s*-CotC-*Cs*CP spores were analysed, as were the effects on the intestinal environment in mice after oral administration of *B.s*-CotC-*Cs*CP spores.

## Methods

### Expression and purification of r*Cs*CP

The coding sequence of *Cs*CP (accession number JN655695), minus the signal sequence, was amplified from a *C. sinensis* cDNA library and cloned into the pET-28a (+) vector. The recombinant plasmid was transformed into *E. coli* BL21 (DE3) (BL21-pET28a-*Cs*CP, abbreviated as BL21-*Cs*CP) and induced by isopropyl β-D-thiogalactopyranoside (IPTG) as described previously [19]. The induced *E. coli* was collected by centrifugation and ultrasonicated to obtain inclusion bodies in sediment. The inclusion bodies were dissolved in phosphate-buffered saline (PBS) containing 2, 4 and 6 M urea, and r*Cs*CP was released into the supernatant. Finally, r*Cs*CP was purified with the His Bind Purification kit (Novagen, Darmstadt, Germany), and gradient elution with 5–400 mM imidazole [15, 19].

### The construction of *B.s*-CotC-*Cs*CP

The coding sequence of coat protein C (CotC) from *B. subtilis* spores, a protein over-expressed on spore surface, was amplified by polymerase chain reaction (PCR) using specific primers (F: 5′-CAT GTC GAC TGT AGG ATA AAT CGT T-3′, R: 5′-CGG AAG CTT GTA GTG TTT TTT ATG C-3′, where the underlined portions are restriction sites for *Sal* I and *Hind* III). The DNA sequence was inserted into the multiple clone site of the pBluescript II SK (−) plasmid [15, 27] after digestion with *Sal* I and *Hind* III. The coding sequence of *Cs*CP was amplified from the previously constructed pET28a (+)-*Cs*CP plasmid using specific primers (F: 5′-CCC AAG CTT AGC AAC ATT CCT GAA TCA G-3′, R: 5′- TAC GAG CTC TCA CAA GAT GAT CGA GGT G-3′) containing restriction sites for *Hind* III and *Sac* I (underlined). The sequence was inserted into pBluescript II SK (−)-CotC plasmid, followed by CotC using *Hind* III and *Sac* I restriction sites, and transformed into *E. coli* DH5α (Promega, Madison, USA). Finally, the CotC- *Cs*CP fusion gene was sub-cloned into a pEB03 shuttle vector after digesting with *Sal* I and *Sac* I. The recombinant plasmid of pEB03-CotC-*Cs*CP was colony-formed in DH5a and easily electro-transformed into *B. subtilis* WB600 to construct *B.s*-CotC-*Cs*CP. Similarly, *B. subtilis* WB600 with the pEB03-CotC plasmid (*B.s*-CotC) was obtained by electro-transformation using specific primers (F: 5′-CAG AAG CTT TGT AGG ATA AAT CGT T-3′, R: 5′-CGC GAG CTC TTA GTA GTG TTT ATG C-3′, restriction sites underlined). All recombinant plasmids above were confirmed by sequencing.

### Preparation of spores and coat proteins

*Bacillus subtilis* WB600 with pEB03-CotC-*Cs*CP was cultured in Difco Sporulation Medium (DSM, BD, Franklin Lakes, USA), and the spores were obtained by the exhaustion method as described previously [28]. After incubation for 24 h, the spores were harvested by centrifugation, treated with 1 mM phenylmethylsulfonyl fluoride (PMSF) plus 4 mg/ml lysozyme for 30 min at room temperature (RT), washed with 1 M NaCl and 1 M KCl, and washed twice with distilled water. Finally, the spores were re-suspended in sterile water and treated in a 68 °C water bath for 1 h to kill the residual WB600 propagules. To extract the coat proteins of spores, the induced spores were treated with sodium dodecyl sulfate (SDS)-dithiothreitol (DTT) extraction buffer (0.5% SDS, 0.1 M DTT, 0.1 M NaCl) at 37 °C for 2 h, washed with 1 M Tris-HCl buffer (pH 8.0) six times, and suspended in 5 ml broken buffer (50 mM Tris-HCl, 0.5 mM EDTA, 1 mM PMSF) and ultrasonicated for 5 min. Coat proteins were collected from the precipitate after centrifugation.

### SDS-PAGE, Western blotting and mass spectrometry analysis

The spores induced at different time points (0 h, 6 h, 12 h and 24 h) and the precipitate/supernatant of the sonicated lysates were analysed by 12.0% SDS-polyacrylamide gel electrophoresis (SDS-PAGE) to evaluate the expression of the CotC-*Cs*CP fusion protein. The separated proteins were transferred onto polyvinylidene fluoride (PVDF) membranes for Western blotting analysis. The membranes were blocked with 5% non-fat milk in phosphate buffer saline containing 0.05% Tween 20 (PBS-T) for 2 h at RT and incubated with rat anti-r*Cs*CP serum (1:600) overnight at 4 °C. After washing three times in PBS-T, the membranes were incubated with HRP-conjugated rabbit anti-rat IgG (1:2000, Sigma, St. Louis, USA) for 1 h at RT and were detected by enhanced chemiluminescence (ECL). Meanwhile, the induced spores containing pEB03-CotC-*Cs*CP or pEB03-CotC, BL21 with pET-28a-*Cs*CP and purified r*Cs*CP from BL21 were comparatively analysed by 12% SDS-PAGE. The corresponding bands of purified r*Cs*CP and CotC-*Cs*CP fusion protein in the polyacrylamide gel were cut out, digested with trypsin and injected onto an ABI 4800 Proteomics Analyzer Matrix-assisted laser desorption ionization time-of-flight mass spectrometer (MALDI-TOF-MS/MS) and Mascot search engine (Matrix Science, London, UK) for protein identification.

### Immunofluorescence and fluorescent confocal microscopy

To confirm whether *Cs*CP displayed on the surface of spores, 200 μl of the treated sporulation suspension was fixed onto slides according to improved methods previously described [14, 15]. Samples were blocked with normal goat serum for 2 h at RT and incubated with rat anti-r*Cs*CP serum (1:200) overnight at 4 °C. Following three PBS washes, Cy3-labeled goat anti-rat IgG (1:400, Invitrogen, Carlsbad, USA) was used to cover the samples smears for 1 h at RT (in dark). The samples were incubated for 3–5 min with a 4′, 6-diamidino-2-phenylindole-(DAPI)-DNA staining solution. Fluorescent images were captured using a Leica DMI4000B fluorescent microscope and a ZEISS LSM 510 META confocal microscope. The induced spores containing pEB03-CotC served as the control.

### Immunogenicity analysis of subcutaneously immunized r*Cs*CP and *B.s*-CotC-*Cs*CP

Female BALB/c mice aged 6–8 weeks were purchased from the Animal Center of Sun Yat-Sen University and raised carefully in accordance with the animal care and the ethical guidelines of the National Institutes of Health.

**Fig. 1:**
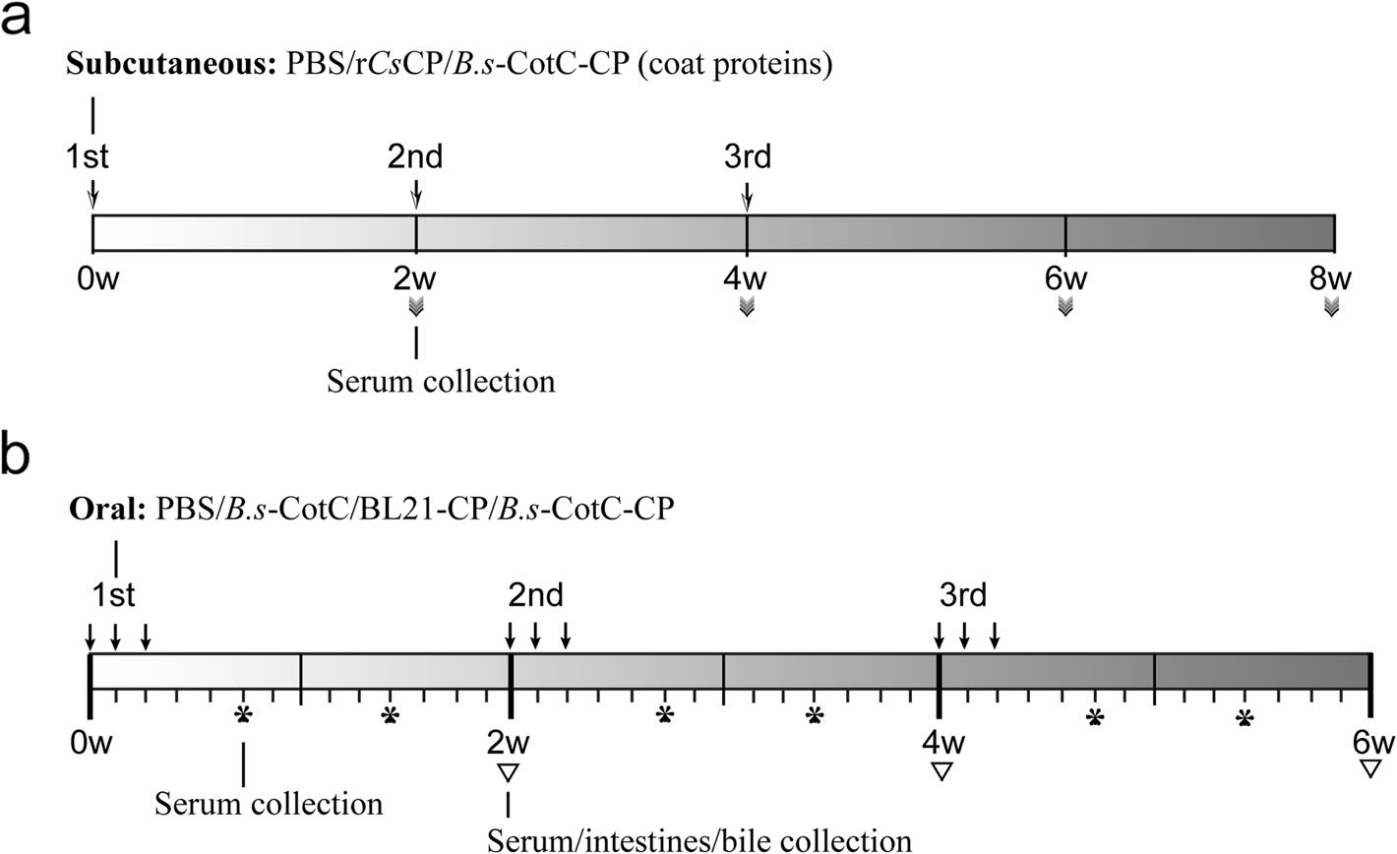
Schematic of the treatment regimen. **a** Subcutaneous immunization of mice with emulsified PBS, r*Cs*CP or spore coat proteins of *B. s*-CotC-*Cs*CP administered three times. Serum samples were collected at 2, 4, 6 and 8 weeks. **b** Oral administration of mice with PBS, spores of *B.s*-CotC or *B.s*-CotC-CP, or BL21-CP three times in total, with continuous gavage for three days each time. Serum, intestine and bile samples were collected every 2 weeks. Additionally, serum samples were collected on days 5 and 10 after each administration. *Abbreviations*: *B.s*-CotC-CP, WB600 containing pEB03-CotC-*Cs*CP; *B.s*-CotC, WB600 harbouring pEB03-CotC; BL21-CP, BL21 harbouring pET28a-*Cs*CP

Each BALB/c mouse was immunized subcutaneously (*s.c*.) with 100 μg r*Cs*CP or the coat proteins of the *B.s*-CotC-*Cs*CP spore, which were emulsified with complete Freund’s adjuvant (Sigma-Aldrich, St. Louis, USA) at the first injection. Control mice were similarly administered with an equal volume of PBS plus adjuvant. Two booster injections at 2-week intervals were performed with 50 μg of proteins or an equal volume of PBS emulsified in incomplete Freund’s adjuvant (Sigma-Aldrich, St. Louis, USA). Anti-sera were collected every 2 weeks for 4 weeks (Fig. 1a) after the final boosting, and antibody titres were determined by ELISA. All anti-sera samples were split and stored at -80 °C.

### Animals, oral immunization and sample collection

Sixty mice were randomly and evenly divided into the following four groups as *B.s*-CotC-*Cs*CP, BL21-*Cs*CP, *B.s*-CotC and PBS. Mice were administered orally by gavage with spores (1 × 10^9^), BL21-*Cs*CP (equivalent *Cs*CP as expressed by the spores), and PBS in a volume of 0.20 ml. Mice were treated three times in total (3 days each time) at biweekly intervals (Fig. 1b).

Serum samples were collected on days 5, 7, 10 and 14 after each administration. Mice were sacrificed two weeks after each oral treatment. The small intestines were isolated and washed 2–3 times with 0.4 ml sterile PBS. The residual mucus was scraped using tweezers. Gallbladders were also collected. The midsection of mouse jejuna (approximately 5–7 mm) was excised and immersed in Bouin’s solution (75% saturated picric acid solution, 25% formalin and 5% glacial acetic acid) for 24 h before histological analysis.

### Detection of *Cs*CP-specific antibodies by indirect ELISA

The levels of IgG, IgG1 and IgG2a in serum were measured by an ELISA assay as described previously [15, 27]. Plates were coated with 5 μg/ml purified r*Cs*CP (0.05 M carbonate-bicarbonate buffer, pH 9.6) at 100 μl per well and incubated at 4 °C overnight. After washing with PBS-T three times, the plates were blocked with 5% skimmed milk for 2 h at 37 °C. After washing routine program, the plates were incubated with serum samples (1:100) for 2 h at 37 °C and subsequently with HRP-conjugated goat anti-mouse IgG (1:2000, Santa Cruz, USA), IgG1 (1:2000, Santa Cruz, USA), or IgG2a (1:2000, Invitrogen, Carlsbad, USA), respectively, for 1 h at RT. Next, 100 μl tetramethylbenzidine (TMB, BD, Franklin Lakes, USA) solution was added to the plates for 10–15 min and stopped by 50 μl of 2 M H_2_SO_4_, and the absorbance was detected at 450 nm. Before changing the incubation solution, the plates were washed with PBS-T 3 times.

Similarly, IgA levels in sera (1:20), intestinal lavage mucus (1:20), and bile (1:100) were also detected. HRP-conjugated goat anti-mouse IgA (1:2000, Southern Biotech, Birmingham, USA) was employed as a secondary antibody.

### Challenge infections and protective efficacy evaluation

Metacercariae of *C. sinensis* were isolated from *Pseudorasbora parva*, captured in Shuangfeng county, Hunan, as previously described [14, 15]. Two weeks after final booster, orally immunized mice (*n* = 5 for each group) were challenged with 30 living *C. sinensis* metacercariae through intragastric administration. All mice were sacrificed at 6 weeks post-challenge infection. Liver tissues were isolated and submitted to histopathological staining.

### Histology staining

After incubation with Bouin’s solution, mouse jejuna from each group were dehydrated, embedded in paraffin wax and sliced into 5-μm sections. The sections were rehydrated with xylene and a gradient ethanol, followed by PBS-T washing. After paraffin removal, the endogenous peroxidase sections were removed by using 3% hydrogen peroxide. The sections were soaked in 0.01 M citrate buffer to retrieve the antigens by high-pressure boiling for 10 min. The slides were blocked with normal goat serum for 1 h at RT, and incubated with goat anti-mouse IgA (1:600, Southern Biotech, Birmingham, USA) overnight at 4 °C. After washing three times with PBS, the slides were incubated with HRP-Protein A (1:4000, Invitrogen, Carlsbad, USA) at RT for 1 h. The immunoreactive signal was developed using 3, 3′-diaminobenzidine (DAB, ZSGB-BIO, China). Finally, the slides were stained with Mayer’s haematoxylin, dehydrated and mounted with neutral balsam. Images were captured under a light microscope (Carl Zeiss, Germany).

For pathological observations, the jejunum sections were stained with routine haematoxylin and eosin (HE). Liver tissue sections from *C. sinensis* metacercariae infected mice were performed with routine Masson staining. All specimens were viewed under a light microscope (Carl Zeiss, Germany). Liver fibrosis of each group was morphologically evaluated by using the Ishak fibrosis score [29].

The mucus production of jejunum was evaluated using alcian blue-periodic acid Schiff reagent (AB-PAS, Baso, China) according to the manufacturer’s protocol. Mucins were observed under a light microscope (Carl Zeiss, Germany).

### Detection of biochemical indices

The serum of each group was collected at week 6. Glutamic pyruvic transaminase/alanine aminotransferase (GPT/ALT) and glutamic oxaloacetic transaminase/aspartate aminotransferase (GOT/AST) were measured using an alanine aminotransferase assay Kit and an aspartate aminotransferase assay Kit, respectively (Jiancheng, China).

### Statistical analysis

Experimental data were presented as the mean ± standard deviation (SD) values. Student’s *t*-test was performed to determine significant differences between groups using SPSS software 13.0 software. *P*-values < 0.05 were considered as statistically significant.

## Results

### Expression and identification of fusion/recombinant protein

The recombinant plasmids pEB03-CotC and pEB03-CotC-*Cs*CP were successfully constructed and sub-cloned into WB600 (Additional file 1: Figure S1). The r*Cs*CP protein was overexpressed in IPTG-induced BL-21 containing pET28a-*Cs*CP, and purified with gradient imidazole (Additional file 1: Figure S1, Additional file 2: Figure S2).

**Fig. 2:**
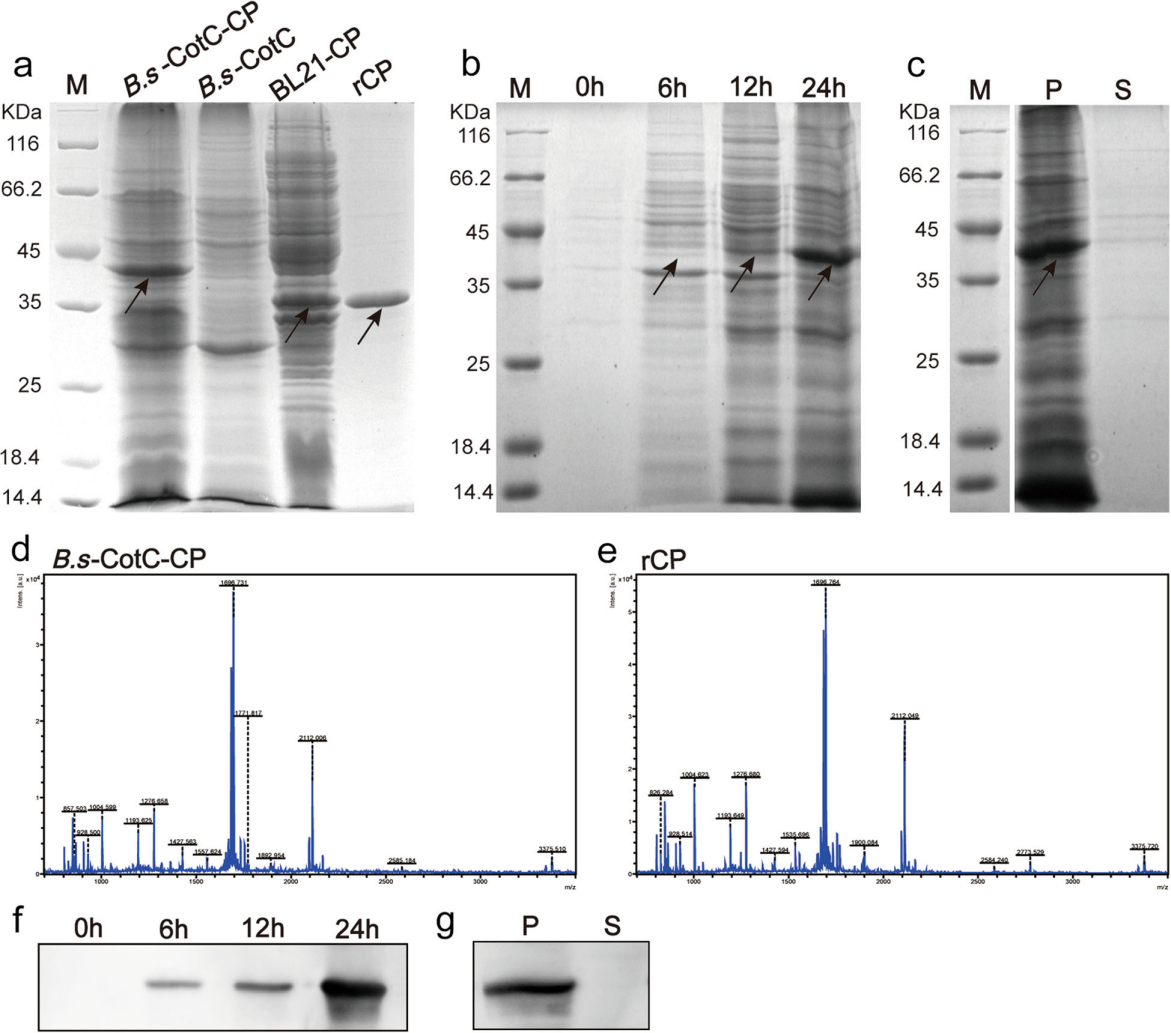
Expression and identification of r*Cs*CP and CotC-*Cs*CP. **a** SDS-PAGE analysis of *Cs*CP expressed in *E. coli* BL21 and *B. subtilis* spores. The molecular mass of CotC-*Cs*CP fusion protein was approximately 43.8 kDa. *Abbreviations*: *B.s*-CotC-CP, WB600 containing pEB03-CotC-*Cs*CP; *B.s*-CotC, WB600 harbouring pEB03-CotC; BL21-CP, BL21 harbouring pET28a-*Cs*CP; rCP, purified r*Cs*CP. **b** The expression of CotC-*Cs*CP fusion protein at different sporulation times by 12% SDS-PAGE. **c** Total spore coat proteins extracted from recombinant spores (pEB03-CotC-*Cs*CP) by SDS-PAGE analysis. **d** Identification of CotC-*Cs*CP fusion protein by MS. **e** MALDI-TOF/TOF-MS analysis of purified r*Cs*CP. **f** Expression identification of CotC-*Cs*CP fusion protein at different sporulation times by Western blotting using rat anti-r*Cs*CP serum. **g** Total coat proteins of pEB03-CotC-CsCP spore recognized by rat anti-r*Cs*CP serum using Western blotting. *Abbreviations*: P, precipitation; S, supernatant

The molecular mass of the CotC-*Cs*CP fusion protein was approximately 43.8 kDa, corresponding to the molecular mass of *Cs*CP (35 kDa) plus CotC (8.8 kDa). As shown in Fig. 2, the CotC-*Cs*CP fusion protein abundantly expressed in the DSM medium induced *B.s*-CotC-*Cs*CP spores, while no matching band was observed in the medium of *B.s*-CotC spores. The expression of the fusion protein gradually increased with incubation time (Fig. 2b). In addition, the spore coat proteins contained large amounts of CotC-*Cs*CP in the precipitation extract but almost no corresponding band was present in the supernatant (Fig. 2c). Western blotting analysis using rat anti-r*Cs*CP serum as the primary antibody showed specific bands at 43.8 kDa in spores samples incubated for 6 h, 12 h and 24 h as well as for coat protein precipitation; however, no bands were observed in propagules and supernatant (Fig. 2f, g).

The mass spectrum analysis of CotC-*Cs*CP and r*Cs*CP is shown in Fig. 2. Both proteins were identified as the cysteine protease of *C. sinensis* and belonged to ProCathepsin L (Table 1).

**Table 1:** MALDI-TOF MS/MS identification results of the proteins from CotC-*Cs*CP and r*Cs*CP

Sample	Protein name	MW (Da)	Protein PI	Protein score	*P*-value	Peptides matched	Swiss Prot	Accession number
CotC-*Cs*CP	*C. sinensis* cysteine protease precursor	35,838	5.11	163	1e^-16^	11	2o6x.1.A	gi|351693702
r*Cs*CP	*C. sinensis* cysteine protease precursor	35,383	5.11	159	2.5e^-16^	11	2o6x.1.A	gi|351693702

### Immunofluorescence analysis of *Cs*CP expressed on the spore surface

**Fig. 3:**
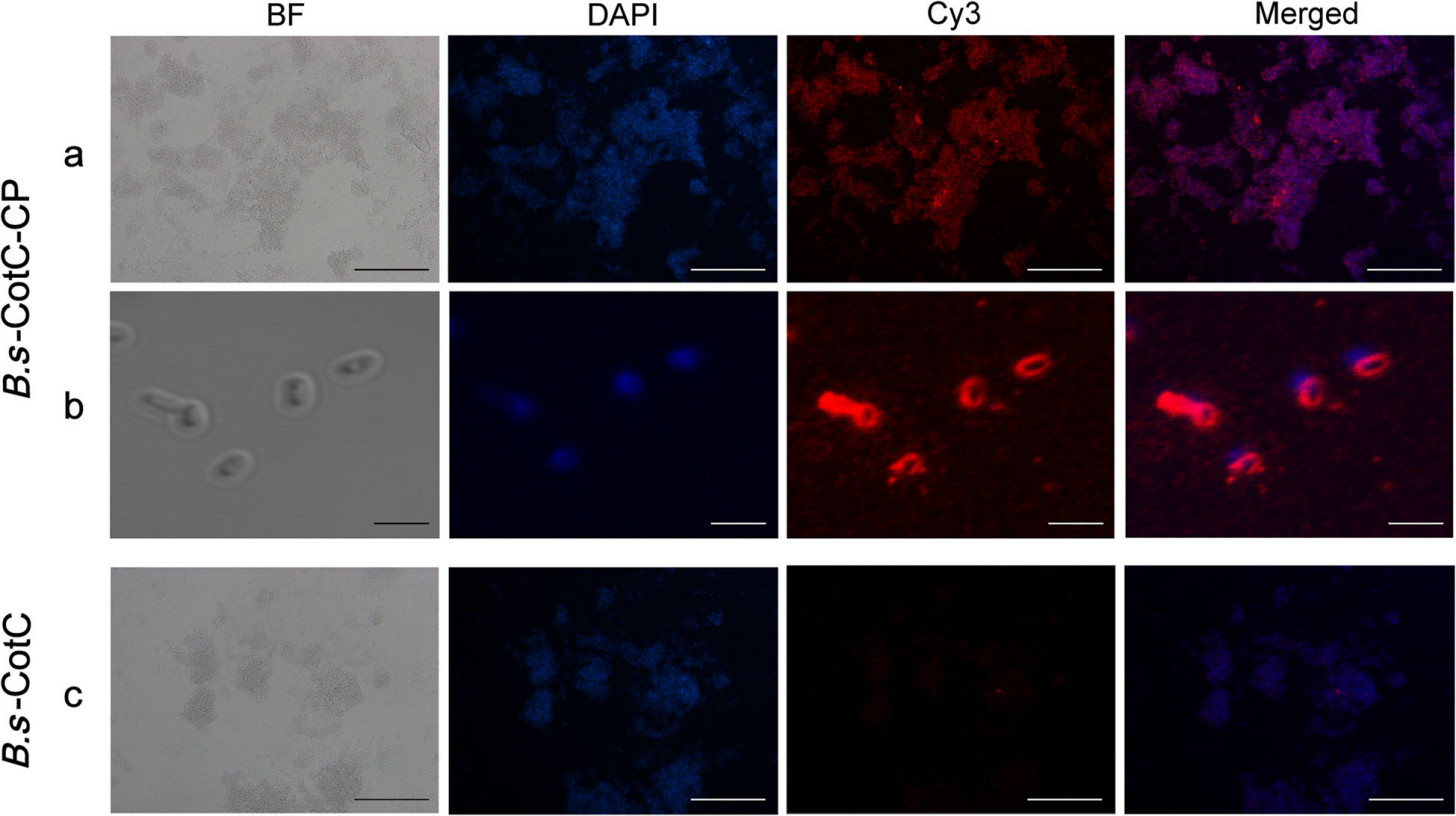
Expression identification of *Cs*CP on the coat of recombinant spores by immunofluorescence. The *B. subtilis* spores with pEB03-CotC-*Cs*CP were observed by immunofluorescence (**a**) and confocal laser microscope (**b**) after incubating with rat anti-*Cs*CP serum and Cy3 labeled goat anti-rat IgG (*red*). The nucleus was stained with DAPI (*blue*). Sporulation CotC strain treated with the same method and both visualized under fluorescent light (**c**). All spores above were observed under bright field (BF) as well. *Abbreviations*: *B.s*-CotC-CP, WB600 containing pEB03-CotC-*Cs*CP; *B.s*-CotC, WB600 harbouring pEB03-CotC. *Scale-bars*: **a**, **c**, 50 μm; **b**, 2 μm

Using the rat anti-r*Cs*CP serum as the primary antibody and followed by Cy3-labelled goat anti-rat IgG as the secondary antibody, immunofluorescence and fluorescent confocal microscopy showed that red fluorescence abundantly distributed surrounded the spores’ coat of *B.s*-CotC-*Cs*CP with a high positive rate (Fig. 3a, b). Almost no red fluorescence was visualized on the *B.s*-CotC spores surface (Fig. 3c).

### Specific IgG and isotypes in serum of subcutaneous immunized mice

**Fig. 4:**
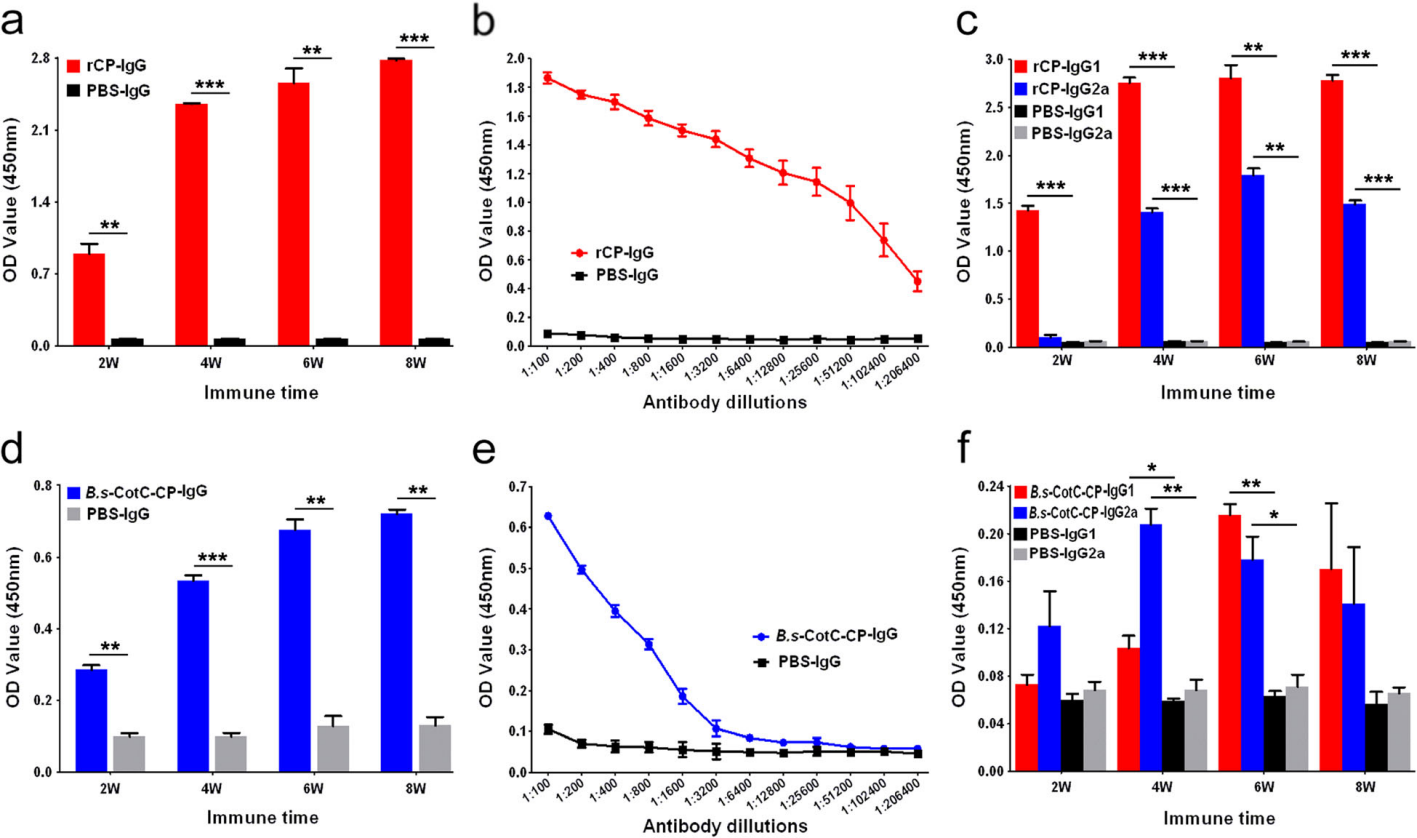
Antibody titres of IgG and isotopes triggered by r*Cs*CP and coat proteins of *B.s*-CotC-*Cs*CP spores *via* subcutaneous immunization route. ELISA evaluation of the *Cs*CP specific IgG **a** and IgG1/IgG2a **c** levels in mouse sera after subcutaneous immunization with r*Cs*CP. **b** Antibody titres of IgG induced by r*Cs*CP at week 6. The levels of *Cs*CP specific IgG **d** and IgG1/IgG2a **f** in the sera of mice subcutaneously immunized with spore coat proteins of *B.s*-CotC-*Cs*CP. Antibody titres of IgG evoked by spore coat proteins of at week 6 were also assayed by ELISA **e**. Data were displayed as the mean ± SD. *P < 0.05; **P < 0.01; ***P < 0.001. *Abbreviations*: *B.s*-CotC-CP, WB600 containing pEB03-CotC-*Cs*CP; *B.s*-CotC, WB600 containing pEB03-CotC; rCP, purified r*Cs*CP

The r*Cs*CP protein provoked markedly higher production of specific IgG compared with the control group only 2 weeks after the first immunization (*t*_(2)_ = 14.72, *P* = 0.005). The significantly higher levels of anti-r*Cs*CP IgG continued rising until four weeks after the last immunization (*t*_(2)_ = 351.27, *P* = 10^-9^). The titre of anti-r*Cs*CP IgG reached 1:206,400 at 6 weeks after immunization (Fig. 4a, b).

After subcutaneous administration of r*Cs*CP, the specific IgG1 level remarkably increased at week 2 (*t*_(2)_ = 49.00, *P* = 10^-9^), while the IgG2a level showed obvious differences at week 4 (*t*_(2)_ = 64.56, *P* = 10^-9^), and both reached their peak at week 6 (Fig. 4c).

The specific IgG and IgG1/IgG2a isotype antibodies were induced by coat proteins extracted from *B.s*-CotC-*Cs*CP spores. The IgG level significantly increased from two to eight weeks after immunization, and the titre of specific IgG reached 1:1600 at week 6 (Fig. 4d, e). The specific IgG1 and IgG2a levels were significantly different compared with those in the PBS group at week 4 and week 6 (Fig. 4f).

### *Cs*CP-specific antibodies in orally administered mice

**Fig. 5:**
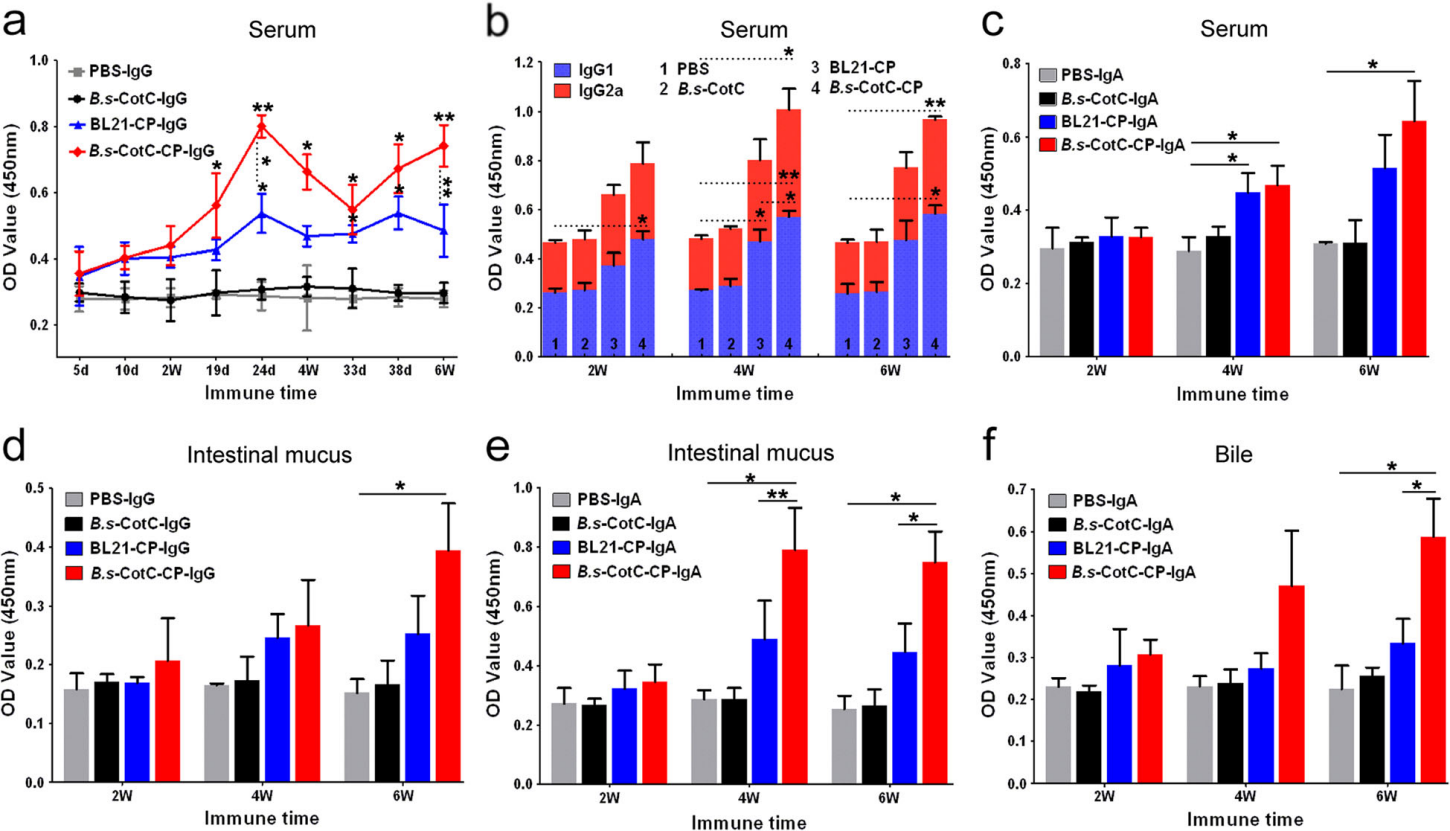
ELISA assay of immune responses triggered by the oral administration of recombinant *B. subtilis* spores. Specific IgG (**a**), IgG1/IgG2a (**b**), and IgA (**c**) levels in sera from mice orally treated with pEB03-CotC-*Cs*CP- or pEB03-CotC-transformed spores, BL21-*Cs*CP and PBS were detected. *Cs*CP-specific IgG (**d**) and sIgA (**e**) levels in intestinal mucous and sIgA level in bile (**f**) were analysed. Data are expressed as the mean ± SD. Statistical significance was analysed by the Student’s *t*-test (*P < 0.05; **P < 0.01). *Abbreviations*: *B.s*-CotC-CP, WB600 containing pEB03-CotC-*Cs*CP; *B.s*-CotC, WB600 containing pEB03-CotC; BL21-CP, BL21 harbouring pET28a-*Cs*CP

The specific IgG level in sera from *B.s*-CotC-*Cs*CP group mice was significantly higher than of the PBS group after day 19 (*t*_(2)_ = 4.66, *P* = 0.043) and reached peak titre on day 24 (*t*_(2)_ = 39, *P* = 0.001). The level was significantly different than the BL21-*Cs*CP group on day 24 and at week 6 (Fig. 5a). Specific IgG1 and IgG2a levels in the *B.s*-CotC-*Cs*CP group were remarkably elevated at weeks 2 and 4, respectively. The levels of IgG1 were significantly different between the *B.s*-CotC-*Cs*CP and BL21-*Cs*CP groups at week 4 (Fig. 5b). Specific IgA levels in the sera of the *B.s*-CotC-*Cs*CP mice were significantly higher than PBS group after week 4 (*t*_(2)_ =5, *P* = 0.038) (Fig. 5c). At some points, the levels of anti-*Cs*CP IgG, IgG1/IgG2a and IgA in serum significantly differed between BL21-*Cs*CP group and PBS group. However, no significant difference was detected between the CotC group and the PBS group (Fig. 5).

Compared with the other three groups, specific IgG levels in intestinal mucus of *B.s*-CotC-*Cs*CP group were relatively higher, and significantly elevated at week 6 (*t*_(2)_ = 4.33, *P* = 0.049) (Fig. 5d). Specific IgA levels in both intestinal mucus and bile of *B.s*-CotC-*Cs*CP group were significantly higher than PBS group at week 4 and week 6, respectively, and showed significant difference compared with BL21-*Cs*CP group at the same time (Fig. 5e, f).

### IgA-secreting cells and mucosubstance in intestine

**Fig. 6:**
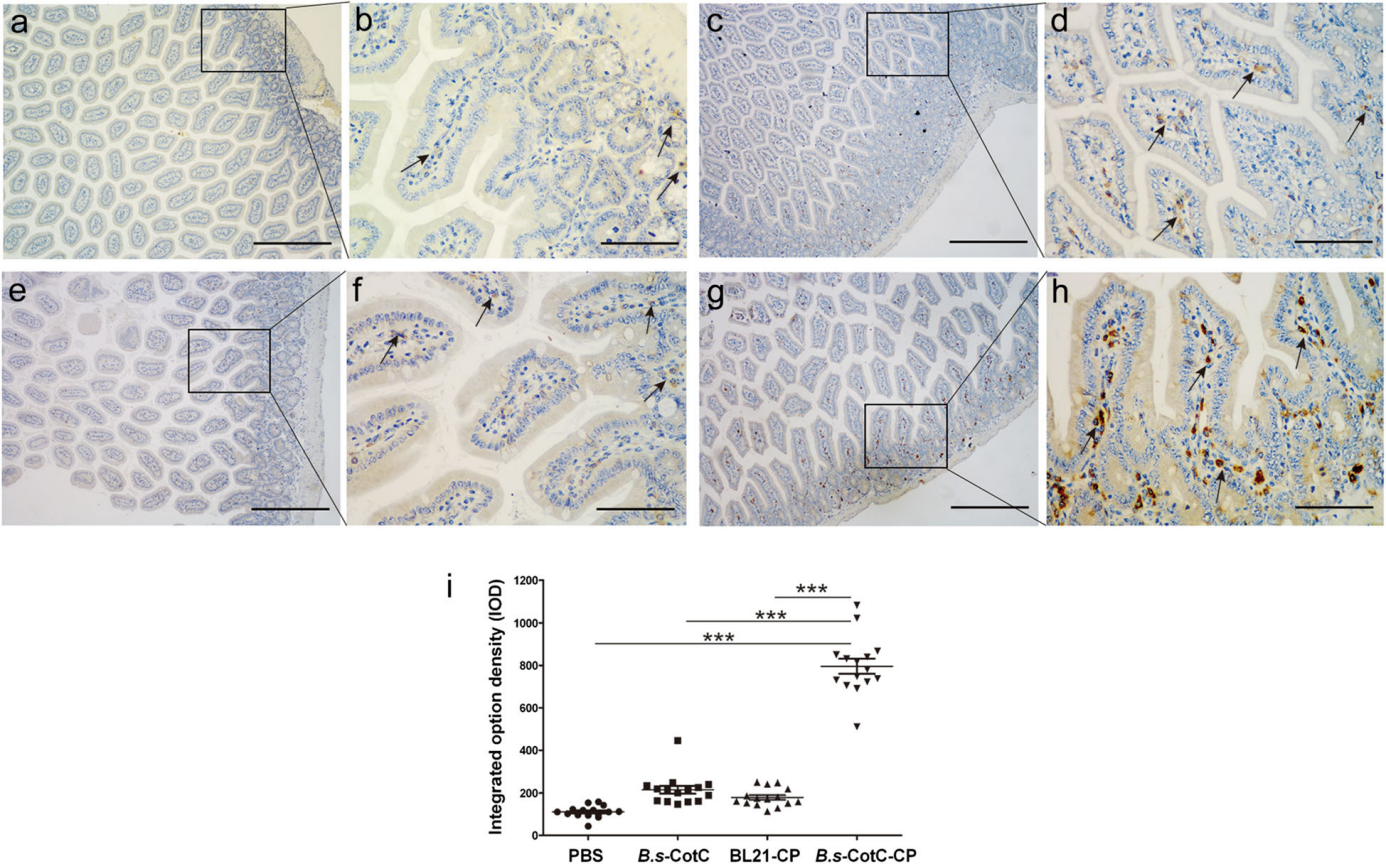
Immunohistochemistry analysis of IgA-secreting cells in the intestinal epithelium of orally immunized mice. IgA-secreting cells were stained dark brown. The jejuna (approximately 5–7 mm) of each group were isolated and submitted to immunohistochemical staining at week 4. Panels (**a**) and (**b**) represent PBS-treated mice. Panels (**c**) and (**d**) represent *B.s*-CotC orally administered mice. Panels (**e**) and (**f**) represent BL21-*Cs*CP gavaged mice. Panels (**g**) and (**h**) represent mice orally administered with spores expressing CotC-*Cs*CP. *Scale-bars*: **a**, **c**, **e**, **g**, 200 μm; **b**, **d**, **f**, **h**, 50 μm. The arrows indicate IgA-secreting cells. **i** Integrated option density (IOD) of IgA-secreting cells. ***P < 0.001

After incubation with goat anti-mouse IgA and HRP-protein A, the sIgA cells in mouse intestine were observed by DAB staining (positive cells were indicated by dark brown). Compared with the other three groups, a large number of dark brown-stained cells were distributed in the enteraden and *lamina propria* regions of the mouse jejunum in the *B.s*-CotC-*Cs*CP group (*P* = 10^-9^; Fig. 6).

**Fig. 7:**
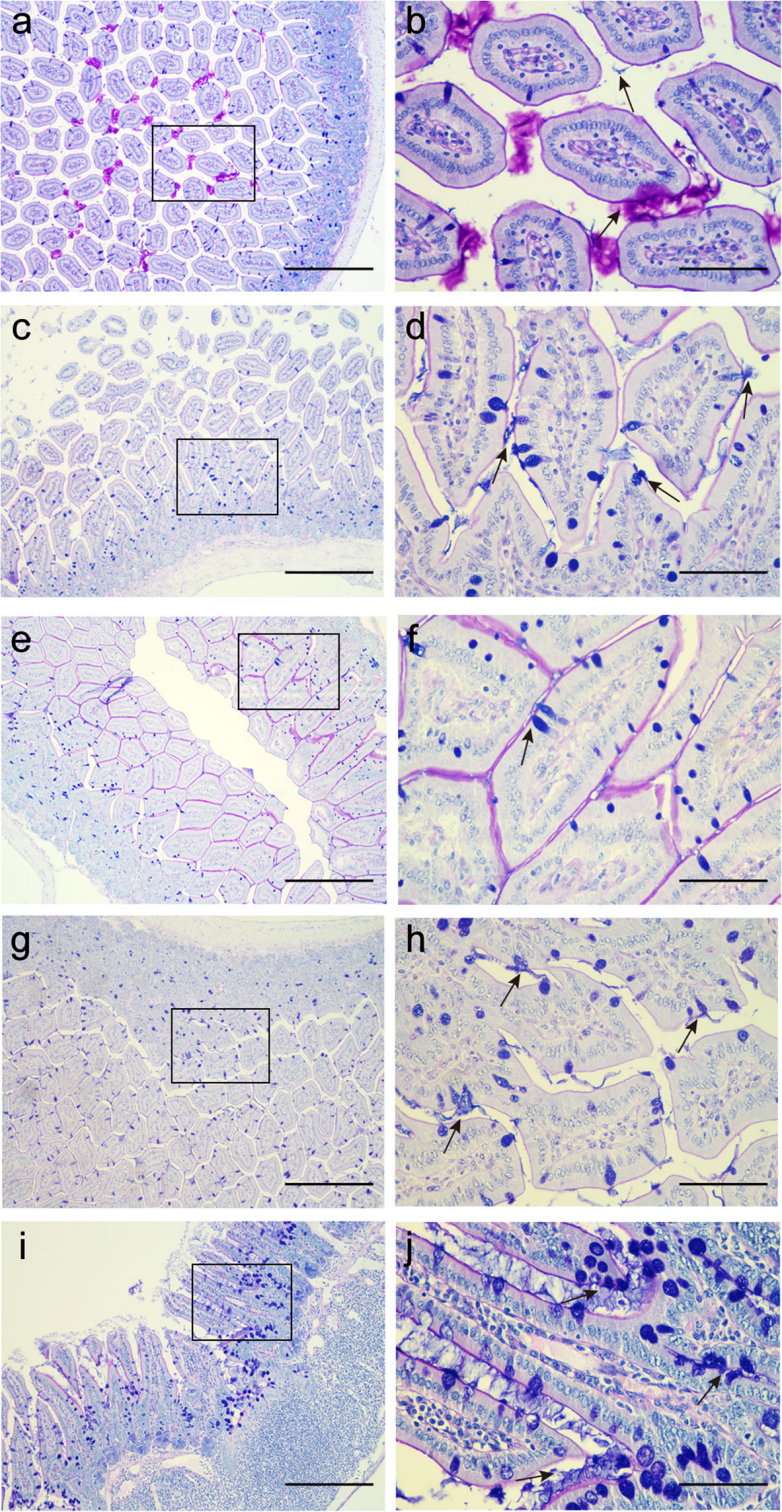
AB-PAS stain of mucins in the intestinal epithelium of oral administration mice. Jejunum tissue sections of each group were collected, fixed, and stained with AB-PAS. Acid mucins were dyed to blue, neutral mucin were dyed red, and the alkaline and neutral mixed mucins were dyed amaranth. Panels **a**-**b**, **c**-**d**, **e**-**f** and **g**-**h** indicate PBS, *B.s*-CotC, BL21-*Cs*CP and *B.s*-CotC-*Cs*CP orally administered groups at week 4, respectively. Panels (**i**) and (**j**) show the *B.s*-CotC-*Cs*CP group treated at week 6. *Scale-bars*: **a**, **c**, **e**, **g**, **i**, 200 μm; **b**, **d**, **f**, **h**, **j**, 50 μm. The arrows indicate acidic mucins secreted by goblet cells

After staining the slides with alcian blue and periodic acid-Schiff, many acidic mucins (marked in blue) were found in the intestines of the *B.s*-CotC and *B.s*-CotC-*Cs*CP groups. A small amount of acidic mucins were also found in the BL21-*Cs*CP group, while copious neutral and alkaline mucus (indicated by amaranth) was present in the PBS group at week 4 (Fig. 7). Two weeks after the last immunization (week 6), a large amount of acidic mucosubstance was released from goblet cells in the *B.s*-CotC-*Cs*CP group (Fig. 7).

### Biochemical index and histological analysis

**Fig. 8:**
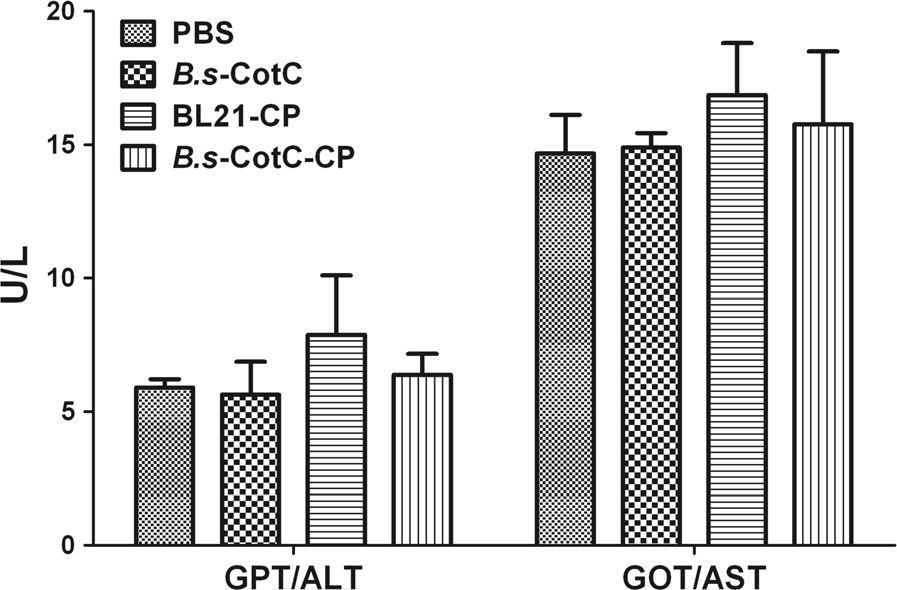
The activity of GPT/ALT and GOT/AST in the serum of oral immunization mice. The serum in PBS, *B.s*-CotC, BL21-*Cs*CP and *B.s*-CotC-*Cs*CP groups was collected at week 6. The levels of GPT/ALT and GOT/AST were analysed. *Abbreviations*: *B.s*-CotC-CP, WB600 containing pEB03-CotC-*Cs*CP; *B.s*-CotC, WB600 of pEB03-CotC; BL21-CP, BL21 harbouring pET28a-*Cs*CP

There was no significant difference in GPT/ALT and GOT/AST levels in serum amongst the groups (Fig. 8). In addition, no inflammatory injury was observed in the intestinal tissues of each group using haematoxylin-eosin staining (Additional file 3: Figure S3).

### Protective efficacy evaluation

**Fig. 9:**
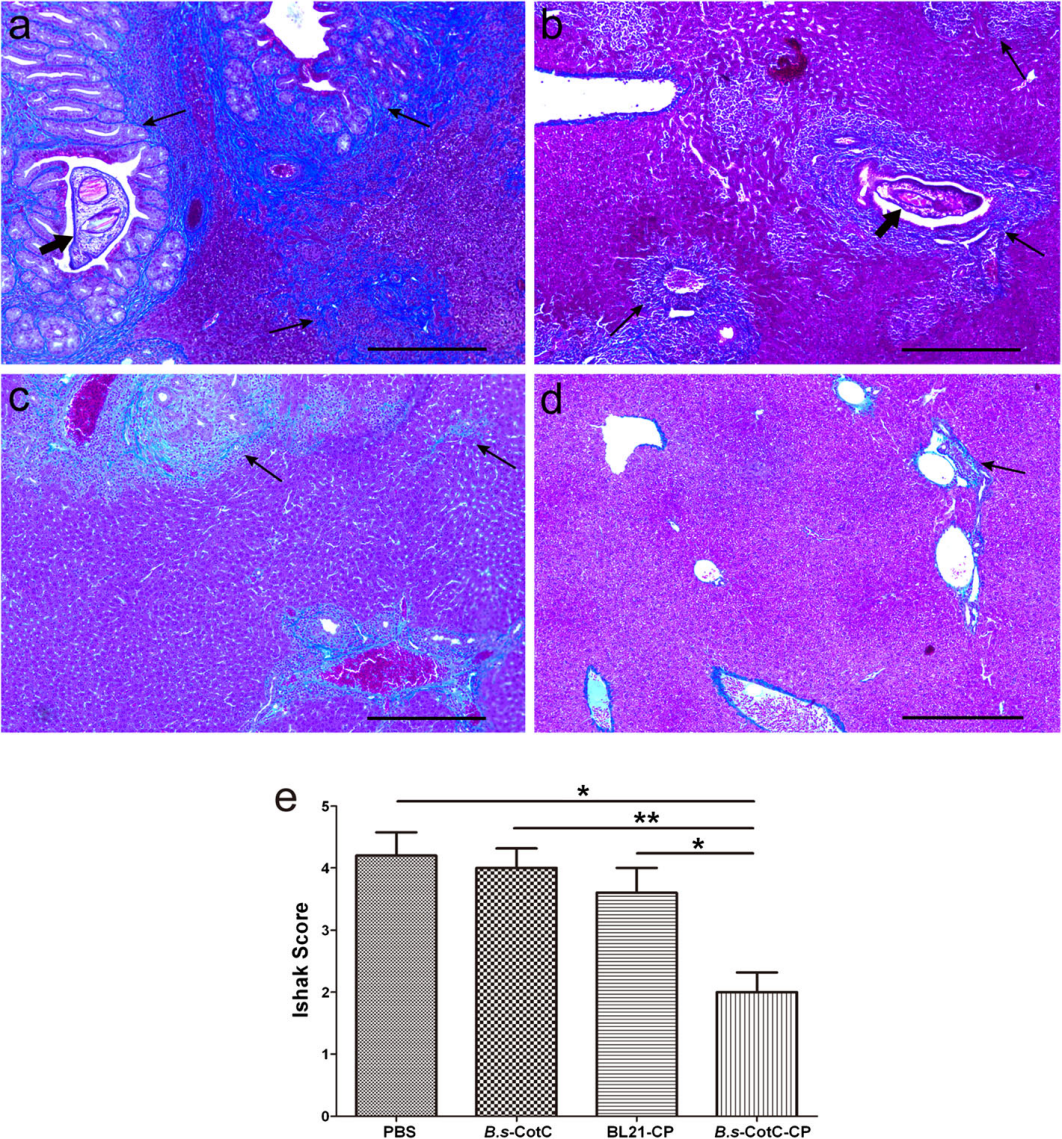
Pathological changes of livers from challenge infection mice. Liver sections of each group were stained with Masson’s trichrome. Collagen fibers are shown in blue. Panels **a**–**d** show mice orally treated with PBS, *B.s*-CotC, BL21-*Cs*CP and *B.s*-CotC-*Cs*CP, respectively. Panel (**e**) provides the statistic of Ishak scores in liver sections from each mouse. *P < 0.05; **P < 0.01. The thick arrows indicate the adult worms of *C. sinensis*, and the thin arrows indicate collagen deposition in livers. *Scale-bars*: 200 μm

Six weeks post-challenge infection, livers of all mice were sent to Masson staining. As shown in Fig. 9, compared with other groups, recombinant spores expressing *Cs*CP administered mice had a lighter collagen deposition in the bile duct or hepatic parenchyma, and exhibited no obvious phenomenon of bile duct hyperplasia. Sections of adult worm were observed in some slides from mice of control groups but not in that from *B.s*-CotC-*Cs*CP group. Ishak scores demonstrated that the degree of hepatic fibrosis could be significantly reduced after orally treated with spores of pEB03-CotC-*Cs*CP (Fig. 9e).

## Discussion

In the current study, we constructed a recombinant plasmid of pEB03-CotC-*Cs*CP that could display original *Cs*CP on the coat of *B. subtilis* WB600 spore. The specific IgG was significantly induced by coat proteins extracted from *B.s*-CotC-*Cs*CP spores after subcutaneous immunization. Compared with control groups, IgA levels in the serum, intestinal mucus and bile of the *B.s*-CotC-*Cs*CP orally treated mice increased remarkably. A larger number of IgA-secreting cells were observed in the enteraden and *lamina propria* regions of the mouse jejunum in the *B.s*-CotC-*Cs*CP group. More acidic mucins and mucosubstance were found in the intestines of the *B.s*-CotC-*Cs*CP group. Moreover, there was no significant difference in the GPT/ALT and GOT/AST levels in the sera from each group. No inflammatory injury was observed in the intestinal tissues of each group too.

The CotC*-Cs*CP fusion protein was abundantly expressed with extended induction time. SDS-PAGE and Western blotting showed that an obvious protein band at approximately 43.8 kDa appeared in the *B.s*-CotC-*Cs*CP sample, and could be specifically recognized by anti-r*Cs*CP rat sera (Fig. 2). In addition, we confirmed that *Cs*CP in the fusion protein presented by *B. subtilis* spores is similar to r*Cs*CP purified from *E. coil* using mass spectrometry (Fig. 2). Immunofluorescence and confocal results clearly revealed that *Cs*CP were enriched around the outer coat of spores (Fig. 3). This observation indicates that the stable spore delivery system was successfully exploited to express *Cs*CP, and the CotC*-Cs*CP fusion protein could directly contact the intestinal tract after oral administration.

In subcutaneously immunized mice, the antibody titres of *Cs*CP specific IgG reached 1:206,400 after three immunizations with r*Cs*CP plus adjuvant (Fig. 4b). It demonstrated that r*Cs*CP elicited high levels of specific antibody rapidly and verified that *Cs*CP was of strong immunogenicity. When mice were administered with r*Cs*CP, the specific IgG1 level in the serum significantly increased after week 2 and continued rising, while IgG2a levels were significantly elevated after week 4 (Fig. 4c). In mice treated with coat proteins extracted from the *B.s*-CotC-*Cs*CP spore, the antibody titres of the specific IgG in serum reached 1:1600 at week 6. IgG1 and IgG2a levels significantly increased at weeks 4 and 6. The IgG2a level at week 4 was higher than that of IgG1, but the levels were inversed at week 6 (Fig. 4d-f). Compared with rCsCP, the specific antibody titres induced by the coat proteins of the *B.s*-CotC-*Cs*CP spore were relatively lower. This result may have occurred because the coat proteins of the *B.s*-CotC-*Cs*CP spore were isolated from the precipitation of spore lysate, which contained many other proteins in addition to *Cs*CP (Fig. 2c). Additionally, an insoluble mixture might trigger a poorer immune response than soluble antigen with subcutaneous administration. The IgG1 antibody response generally represents helper T (Th) 2-mediated humoral immunity, while IgG2a represents Th1-mediated immunity [30, 31]. Our results showed that both the r*Cs*CP and coat proteins of the *B.s*-CotC-*Cs*CP spore could elicit Th1/Th2 combined immune responses in mice, but r*Cs*CP induced a Th2 dominant response. There was no question as to the coat proteins of the *B.s*-CotC-*Cs*CP spore. Collectively, these data demonstrated that *Cs*CP delivered by *B. subtilis* spores maintain the immunogenicity and effectively induce an immune response in mice.

*Clonorchis sinensis* metacercariae excyst in the duodenum of the definitive host and develop into larvae within a few minutes. Larvae rapidly migrate into bile ducts, and further maturate into adults [1, 2], so that during the invasion and parasitism, both the intestinal tract and the bile duct are important places for the development and growth of *C. sinensis*. This behaviour suggests that intervention measures applied in these two locations may be effective against *C. sinensis* infection. Cysteine proteases mainly distribute to the excretory bladder and the excretory granules of metacercariae. The cysteine protease family is one of the most abundantly expressed protease families in many digenetic trematode parasites (e.g. *C. sinensis*, *Schistosoma japonicum*, *Clinostomum complanatum*, *Fasciola hepatica* and *Euclinostomum heterostomum*) [18]. Moreover, cysteine protease is a key intrinsic endogenous enzyme that participates in the process of metacercariae excystment, invasion and immune modulation [18, 26, 32, 33]. Collectively, cysteine protease is considered to be a potential candidate molecule for the development of oral vaccine against *C. sinensis* infection.

Spores, the quiescent cell form of *B. subtilis*, are an ideal vehicle for delivery of heterologous antigens in the gastrointestinal tract because they are probiotics and resistant to extreme environments. They have previously served as effective adjuvants as well [15, 16, 34]. *Bacillus subtilis* spores (approximately 1.2 μm in length) are well uptaken by M cells, transported into Peyer’s patches, processed by antigen-presenting cells, presented to B cells or T cells, and are transported to other gut-associated lymphoid tissues (GALTs) and systemic lymphoid tissues [14, 35]. During this process, a series of antigen-specific immune responses are induced and abundant immune globulins are secreted by plasma cells [36]. As sIgA is the most abundantly produced immunoglobulin in the mammal mucosal system, it can serve as the first line of defence in protecting the intestinal epithelium from pathogenic microorganisms and enteric toxins [37, 38]. In addition, sIgA promotes the clearance of pathogenic microorganisms and antigens by blocking their access to epithelial receptors, entrapping them in mucus, and facilitating their removal by peristaltic and mucociliary activities [38]. Our results show that *Cs*CP recombinant spores evoked plenty of specific sIgA in intestinal mucosa and bile (Fig. 5e, f). A large number of IgA-secreting cells were observed in the jejunum of *B. subtilis* spores in orally administered mice, especially in the *B.s*-CotC-*Cs*CP group (Fig. 6). IgA-secreting cells were primarily intestinal epithelial and *lamina propria* lymphocytes distributed in the enteraden and intestinal villus (Fig. 6). In addition, specific IgG arouse in intestinal mucus of *B.s*-CotC-*Cs*CP group (Fig. 5d). Moreover, the levels of IgG, IgG1/IgG2a and IgA in the serum of the *B.s*-CotC-*Cs*CP group were significantly higher than the control group (Fig. 5a-c). These results demonstrated that both local mucosal and systemic humoral immune responses were triggered by the oral administration of *B.s*-CotC-*Cs*CP. Data also confirmed that the spores carried antigens that could trigger higher levels of specific antibody and be more suitable than *E. coli* for the development of oral vaccine vehicles carrying heterologous proteins.

*Bacillus subtilis* spores are probiotic microorganism that are used as a food supplement and can promote intestinal health (e.g. aiding in digestion and being beneficial for the balance of intestinal microbiota and regulation of intestinal mucus environment) [10, 39]. The mucus layer overlaying the epithelium secreted by the goblet cells of the gastrointestinal tract serves as the first line of defence against physical and chemical injury from luminal contents and pathogens and provides a natural habitat for commensal microbiota [40–42]. Mucins, the major component of mucus, can be broadly categorized into neutral and acidic chemotypes. High levels of acidic mucins such as sialomucin and sulfomucin reportedly coincide with maturation of intestinal barrier function and have been implicated in the protection of the mucosa from pathogens [43, 44]. Microbiota and microbial products, meanwhile, can modulate the synthesis and secretion of mucins, either by directly activating diverse signalling cascades or through bioactive factors generated by epithelial and *lamina propria* cells [42]. In our experiments, *B. subtilis* spores could provoke an increase in acidic intestinal mucus (Fig. 7). Thus, *B. subtilis* spores may enhance resistance to pathogens, and guarantee intestinal health.

It has been documented that *B. subtilis* spores are safe food probiotics. No signs of toxicity or virulence were found *in vivo* assessments, and no side effects appeared in the liver function of the rats after oral treatment [10, 16]. Our findings also confirmed that there was no damage to the liver or intestine of mice after oral administration of *B. subtilis* spores (Fig. 8; Additional file 3: Figure S3). Challenge experiment verified that oral administration of *B. subtilis* spores expressing *Cs*CP could effectively fight against metacercaria *C. sinensis* infection by reducing the process of liver fibrosis in mice (Fig. 9).

## Conclusions

We confirmed the excellent immunogenicity of *Cs*CP and *B. subtilis* spores as an oral delivery vehicle that maintained their original immunogenicity by displaying *Cs*CP on the spore surface. Both local and systemic specific immune responses were elicited by the oral administration of *B. subtilis* spores expressing *Cs*CP. Moreover, the spores could effectively promote intestinal health by inducing the secretion of acidic mucins with no side effects to the liver or intestine. Effective protection against *C. sinensis* was triggered after oral administration of spores expressing *Cs*CP. This study is a cornerstone for the development of antiparasitic agents or vaccines against clonorchiasis based on *B. subtilis* spores expressing *Cs*CP on the surface.

## Additional files

Additional file 1
**Figure S1.** Genetic engineering of *B. subtilis* spores and *E. coil* expressing *Cs*CP. (DOC 977 kb)
Additional file 2
**Figure S2.** Prokaryotic expression and purification of the recombinant *Cs*CP. (DOC 2128 kb)
Additional file 3
**Figure S3.** Histopathological features of the jejuna from spores immunized mice. (DOC 5180 kb)

## Abbreviations

*B. s.*: *Bacillus subtilis*
CCA: Cholangiocarcinoma
CotC: Coat protein C, one of coat proteins of *B. subtilis* spore
*Cs*CP: Cysteine protease of *C. sinensis*
DALYs: Disability adjusted life years
ESPs: Excretory-secretory products.
GOT/AST: Glutamic oxaloacetic transaminase/aspartate aminotransferase
GPT/ALT: Glutamic pyruvic transaminase/alanine aminotransferase
r*Cs*CP: Recombinant *Cs*CP protein
