# Supplementary material for: The immunological characteristics and probiotic function of recombinant Bacillus subtilis spore expressing Clonorchis sinensis cysteine protease
Source: Parasit Vectors. 2016 Dec 19;9:648. doi: 10.1186/s13071-016-1928-0 (PMC5170900; doi:10.1186/s13071-016-1928-0)
Supplement: Additional file 1 — Figure S1. Genetic engineering of B. subtilis spores and E. coil expressing CsCP. (DOC 977 kb) [file 13071_2016_1928_MOESM1_ESM.doc]

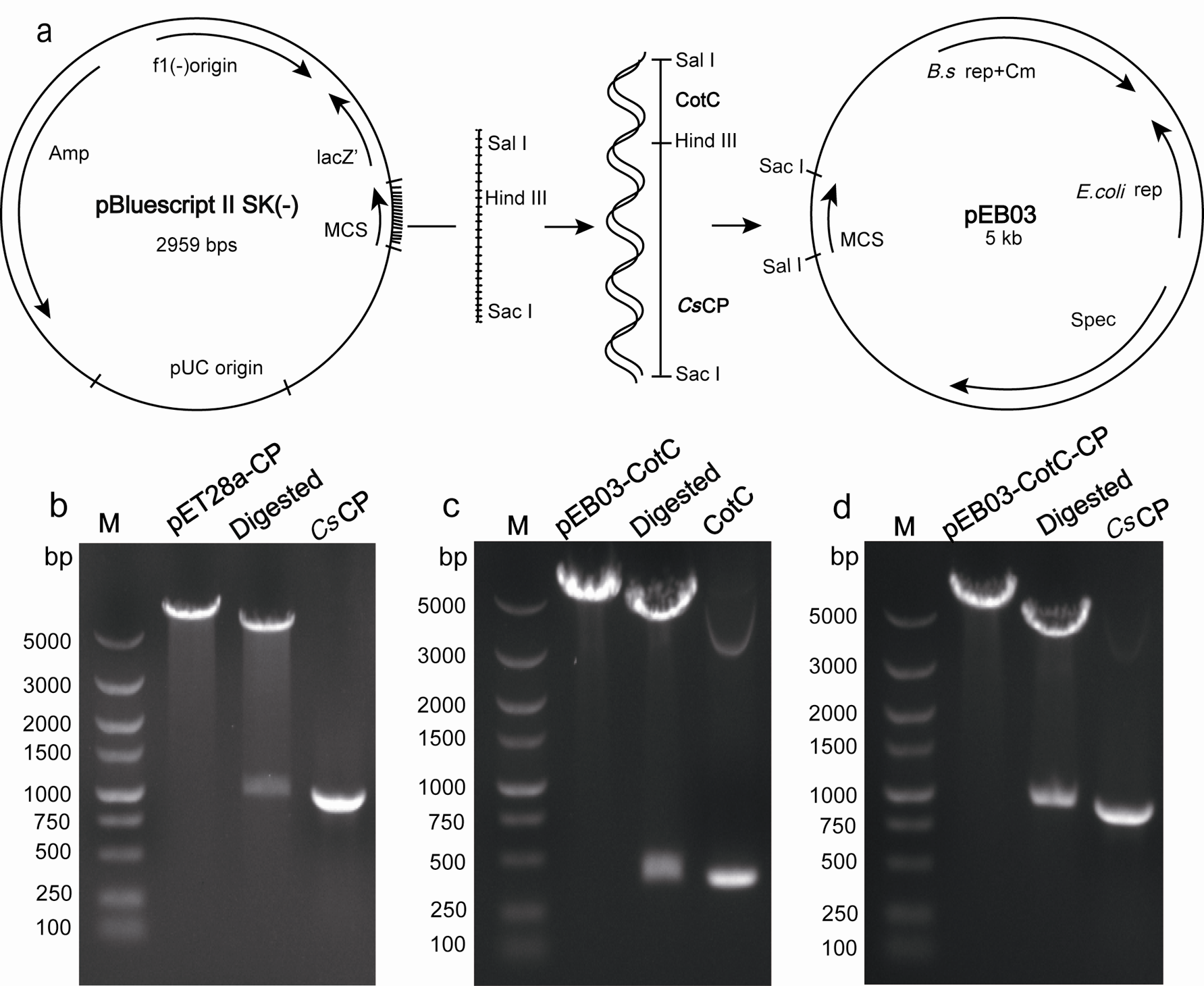


**Figure S1.** Genetic engineering of *B. subtilis* spores and *E. coil* expressing *Cs*CP. **a** Construction strategy of recombinant plasmid of pEB03-CotC-CsCP. **b** Full coding sequence of CsCP was cloned into pET28a(+) vector. **c** Full coding sequence of CotC was cloned into the *E. coli* /*B. subtilis* shuttle vector pEB03. **d** Construction of recombinant pEB03-CotC-*Cs*CP plasmid
