# Supplementary material for: The immunological characteristics and probiotic function of recombinant Bacillus subtilis spore expressing Clonorchis sinensis cysteine protease
Source: Parasit Vectors. 2016 Dec 19;9:648. doi: 10.1186/s13071-016-1928-0 (PMC5170900; doi:10.1186/s13071-016-1928-0)
Supplement: Additional file 2 — Figure S2. Prokaryotic expression and purification of the recombinant CsCP. (DOC 2128 kb) [file 13071_2016_1928_MOESM2_ESM.doc]

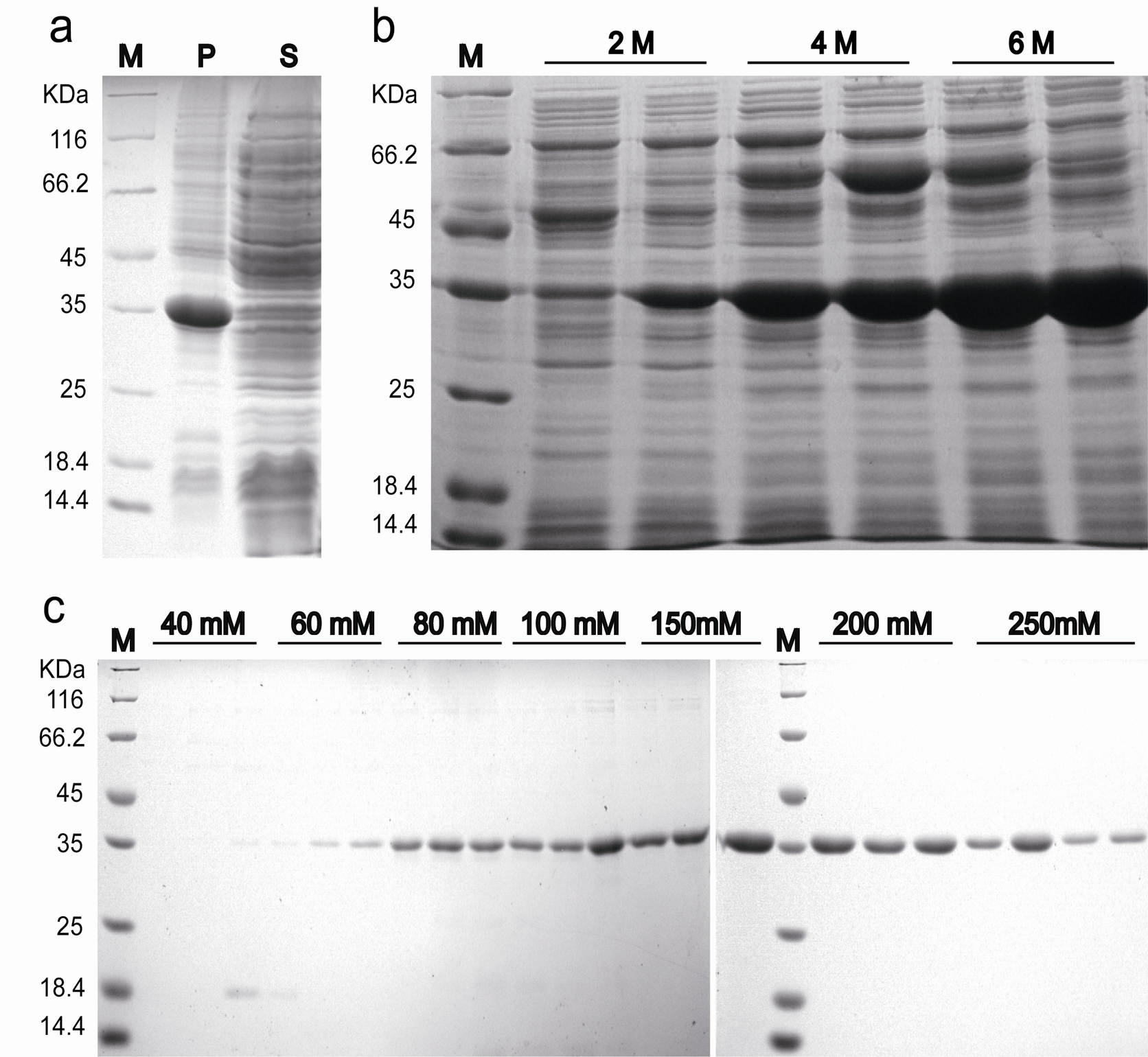


**Figure S2.** Prokaryotic expression and purification of the recombinant *Cs*CP. **a** Collection of the ITPG induced BL21-*Cs*CP and 12% SDS-PAGE analysis of the ultrasonic treated precipitation and supernatant. P: precipitation, S: supernatant. **b** Denaturation of inclusion bodies containing r*Cs*CP and collection of the supernatant from inclusion bodies solubilized in 2 M, 4M and 6 M urea. **c** The r*Cs*CP was purified from BL21 using His-tag resin and eluted by gradient concentration of imidazole
