# Supplementary material for: The immunological characteristics and probiotic function of recombinant Bacillus subtilis spore expressing Clonorchis sinensis cysteine protease
Source: Parasit Vectors. 2016 Dec 19;9:648. doi: 10.1186/s13071-016-1928-0 (PMC5170900; doi:10.1186/s13071-016-1928-0)
Supplement: Additional file 3 — Figure S3. Histopathological features of the jejuna from spores immunized mice. (DOC 5180 kb) [file 13071_2016_1928_MOESM3_ESM.doc]

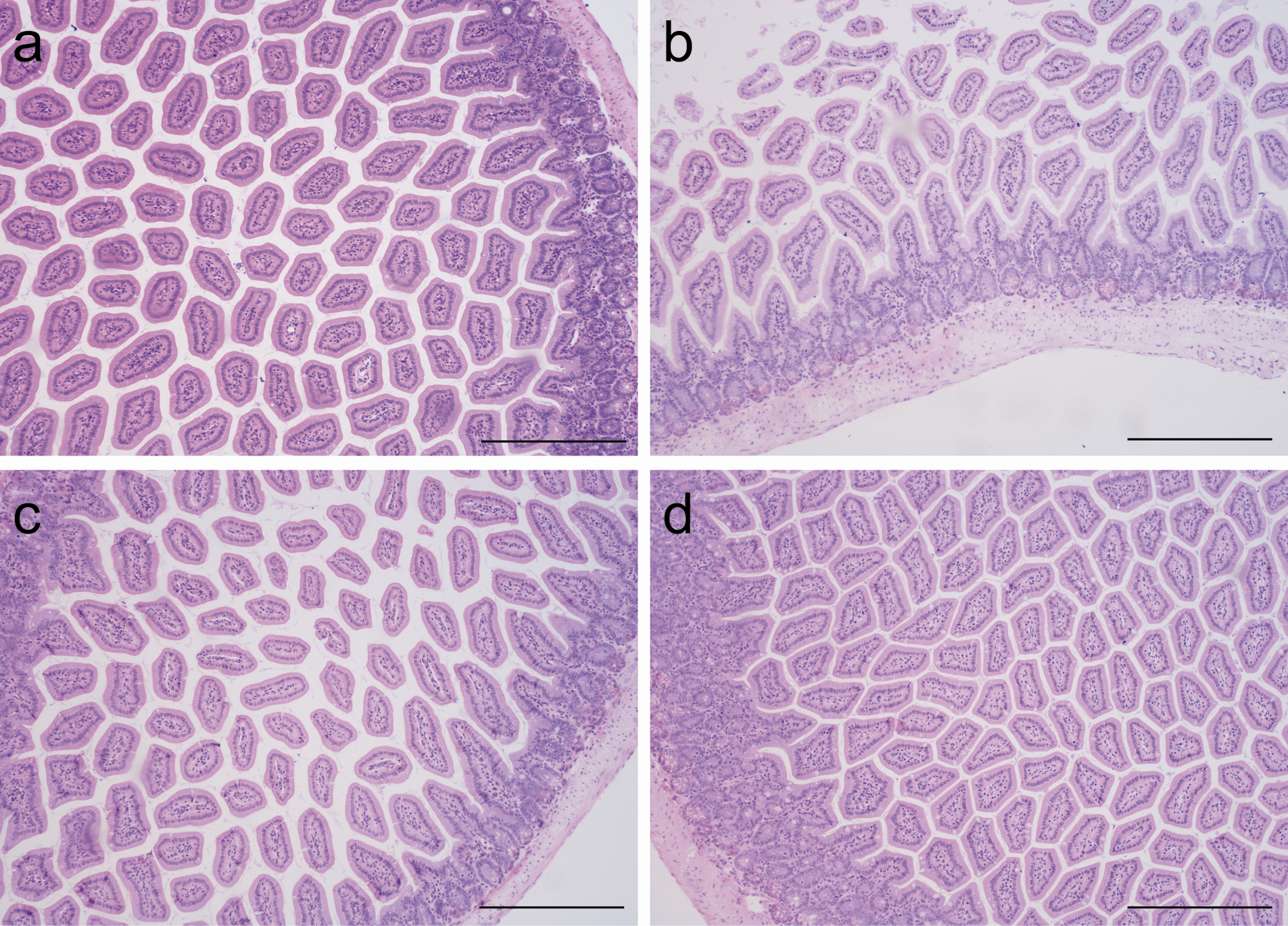


**Figure S3.** Histopathological features of the jejuna from spores immunized mice. **a** PBS group. **b** *B.s*-CotC group. **c** BL21-*Cs*CP group. **d** *B.s*-CotC-*Cs*CP group
